# A Novel *Bacillus safensis*-Based Formulation along with Mycorrhiza Inoculation for Controlling *Alternaria alternata* and Simultaneously Improving Growth, Nutrient Uptake, and Steviol Glycosides in *Stevia rebaudiana* under Field Conditions

**DOI:** 10.3390/plants11141857

**Published:** 2022-07-15

**Authors:** Jai Prakash, Dilfuza Egamberdieva, Naveen Kumar Arora

**Affiliations:** 1 Department of Environmental Science, School of Earth and Environmental Sciences (SEES), Babasaheb Bhimrao Ambedkar University (A Central University), Vidya Vihar, Raebareli Road, Lucknow 226 025, Uttar Pradesh, India; jpmicrobio@gmail.com; 2 Faculty of Biology, National University of Uzbekistan, Tashkent 100174, Uzbekistan; dilfuza.egamberdieva@zalf.de

**Keywords:** *Bacillus*, mycorrhiza, biocontrol, formulation, *Alternaria*, nutrient uptake, *Stevia rebaudiana*

## Abstract

The excess use of chemicals by farmers in the agroecosystems degrades soil quality, disturbs soil ecology, and increases soil salinity and health hazards in humans. *Stevia rebaudiana* is an important medicinal and aromatic crop whose leaves contain steviol glycosides (SGs). The *Bacillus safensis* NAIMCC-B-02323 strain STJP from the rhizosphere of *S. rebaudiana* producing salicylic acid (16.80 µg/mL), chitinase (75.58 U/mL), β-1,3-glucanase (220.36 U/mL), and cellulase (170 U/mL) was taken as a plant growth-promoting rhizobacteria (PGPR). The cell-free supernatant (CFS) from strain STJP showed significant biocontrol activity against *Alternaria alternata* (80%), suggesting the protective role of extracellular metabolite(s) against phytopathogens. Paneer whey-based bioformulation (P-WBF) was developed to exploit *B. safensis* STJP to enhance the growth, nutrient uptake, soil properties, stevioside content, and SGs biosynthesis in *S. rebaudiana* under an *A. alternata*-infested field. The combined treatment of P-WBF and mycorrhiza (*Glomus fasciculatum* ABTEC) significantly enhanced plant growth parameters after 90 days, in comparison with control. The symbiotic action (P-WBF and mycorrhiza) displayed much better results in terms of chlorophyll a and b (improved by 132.85% and 39.80%, respectively), protein (by 278.75%), flavonoid (by 86.99%), carbohydrate (by 103.84%), antioxidant (by 75.11%), and stevioside (by 120.62%) contents in plants as compared to the untreated set. Further, the augmentation of potassium (by 132.39%), phosphorous (by 94.22%), and zinc (by 111.11%) uptake in plant tissues and soil was also observed by the application of P-WBF and mycorrhiza. The expression of UGT74G1 and UGT85C2 genes related to SG biosynthesis was upregulated (2.7- and 3.2-fold, respectively) in plants treated with P-WBF and mycorrhiza as further confirmed by the accumulation of SGs. The results suggest that the application of P-WBF and mycorrhiza not only provides an ecofriendly and sustainable solution to improve stevioside content in *S. rebaudiana* by a nutrient-linked mechanism but also paves the way to enhanced production of stevioside.

## 1. Introduction

An important medicinal, aromatic, and industrial crop, *Stevia rebaudiana* is a substitute for sugar with broad medicinal properties and superior flavor outline and is also of agronomic importance [1]. *S. rebaudiana* contains various vitamins and minerals, such as Fe, Si, Co, Mn, Ca, Mg, Se, Ti, and Zn, and has been explored for its curative practices as hepatoprotective [2], antihyperglycemic [3], antitumor [4], anti-inflammatory [5], and immunomodulatory [6]. In addition, it is well known as a natural sweetener due to its low glycemic index, which is dependent on key alkaloids known as steviol glycosides (SGs), and thus there is a huge demand for it in the agro, food, beverage, and pharmaceutical industries [7]. The two main SGs are stevioside and rebaudioside-A, which are 300 and 420 times sweeter than sucrose and have a longer shelf life [7]. The biosynthesis of SGs shows the plastidial 2-C-methyl-D-erythritol- 4-phosphate pathway (MEP pathway), which is similar to that of gibberellin [8]. It has many steps in the MEP pathway; among them, the last step of SG biosynthesis is present in the cytosol of a plant cell, catalyzed by uridine diphospahate-(UDP) glycosyltransferases (UGTs) [9]. Among all UGTs, three main genes, namely UGT85C2, UGT74G1, and UGT76G1, have been identified for SG biosynthesis [10]. UGT85C2 catalyses the addition of C13-glucose to steviol, resulting in the formation of steviolmonoside, which is then glycosylated into steviolbioside. The glycosylation of steviolbioside’s C-4 carboxylic acid by the UGT74G1 enzyme results in the synthesis of stevioside [7]. Afterward, rebaudioside-A is formed by the addition of a D-glucose molecule of stevioside, catalyzed by the UGT76G1 enzyme [11]. There are a few reports available on the impact of biomolecules, such as gibberellin, salicylic acid (SA), and chitosan, on gene expression associated with SGs biosynthesis in stevia crop [10,11] Some work has been done on the direct growth promotion of stevia using arbuscular mycorrhizal fungi (AMF) [12]. However, there is no research available concerning the role of plant growth-promoting rhizobacteria (PGPR) in the regulation of SGs, metabolite content, growth, biochemical aspects, and management of phytopathogens of stevia. 

Various abiotic and biotic stresses, such as the nutrient deficiency and soil-dwelling phytopathogens, not only hamper the growth of crops, including *S. rebaudiana*, but also have an impact on soil health by disturbing soil quality, soil habitat, nutrient pool, and indigenous microbial diversity [13,14]. Soil-dwelling pathogens are a major problem worldwide and are the key factors for the reduction of crop yields [15]. The deadly fungal phytopathogen *Alternaria alternata* causes havoc in several crops, including *S. rebaudiana,* by leaf spot and leaf blight diseases [16]. *Alternaria* spp. are opportunistic pathogens that cause leaf spot, rot, and leaf blight diseases in a variety of crops, including *S. rebaudiana*, with production losses of up to 80% in affected fields. The disease is identified in *S. rebaudiana* by the appearance of necrotic lesions or the dark brown coloration of the leaf. After infection and during pathogenesis, when fungal metabolites damage the plant during its active phase of growth, symptoms of disease arise [15,16]. Various methods (crop rotation, cultural practices, disease-resistant varieties, and chemical pesticides) are available for the management of phytopathogens and soil improvement [17], but there is no single method that is satisfactory for the control of disease-related plants and to enhance productivity. However, the use of pesticides not only restricts medicinal plant cultivation due to their direct consumption by humans but also disrupts soil ecology and degrades soil properties [18]. A change from chemical-based toward ecofriendly methods is required to maintain the environmental sustainability. PGPR are well known as biostimulants, and their roles are mainly considered in plant growth promotion [18,19]. Among PGPR, *Bacillus* is one of the most effective genera for enhancing the growth and yield of crops under biotic and abiotic conditions due to their spore-forming property and production of several metabolites [20,21]. AMF are a type of obligate biotroph that form mutualistic symbiotic associations with the roots of plants [22]. AMF are also commonly used as biofertilizers for enhancing growth and crop productivity [23]. In addition to plant growth, AMF provide tolerance to host plants against several fungal pathogens and help in survival under stressful conditions [22].

However, no reports are available to understand the simultaneous effect of PGPR and AMF on growth, metabolite content, nutrient status, and plant growth attributes of *S. rebaudiana* under natural conditions, where the plant is exposed to stresses such as soil-dwelling phytopathogens and deficiency of soil nutrients The present study was focused to augment the stevioside accumulation in *S. rebaudiana* by using PGPR alone and in combination with AMF. The effect of PGPR and AMF on growth parameters, such as shoot and root length, fresh weight (FW) and dry weight (DW), number of leaves, chlorophyll, antioxidant, flavonoid, and nutrient uptake in plants; and soil health, was checked. In addition, the relative highest transcript profiles of candidate genes involved in the biosynthesis of SGs in *S. rebaudiana* was investigated after the application of PGPR and AMF. The developed novel method may prove to be a valuable asset for farmers involved in the organic farming of *S. rebaudiana*.

## 2. Results

### 2.1. Biocontrol Traits

Bacterium STJP produced salicylic acid (16.80 µg/mL), chitinase (75.58 U/mL), β-1, 3-glucanase (220.36 U/mL), and cellulase (170 U/mL) activities. However, it did not produce hydrogen cyanide (HCN). 

**Figure 1:**
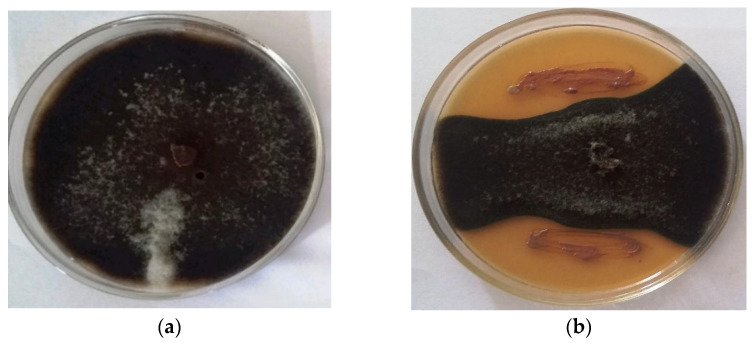
Control plate of *A. alternata* showing healthy mycelia (control) (**a**). Inhibition of *A. alternata* growth by CFS of isolate STJP (**b**).

Cell-free supernatant (CFS) of isolate STJP showed better inhibitory activity against *A. alternata* in comparison to cell suspension (CS). The CS recorded 32.5% inhibition of fungal growth, while the CFS of isolate STJP showed an 80% inhibitory effect on the fungal pathogen (Figure 1).

### 2.2. Impact of Bioformulation on Growth of S. Rebaudiana

*B. safensis* STJP (Paneer whey-based bioformulation (P-WBF)) positively affected the plant growth parameters as observed after 30, 60, and 90 days after sowing (DAS) of seedlings in the field (Supplementary Figure S1). Among all treatments, P-WBF plus mycorrhiza recorded the maximum increment in vegetative parameters as compared to control (Table 1).

P-WBF and mycorrhiza (combined) treatment enhanced FW and DW by 91.36% and 78.94%; 73.41% and 104.82%; and 136.30% and 184.22% after 30, 60, and 90 DAS of seedlings, in comparison with control plants, respectively (Table 1). Only P-WBF-treated plants also showed significant enhancement in FW (72.99%, 59.10%, and 92.64%) and DW (53.07%, 81.97%, and 131.03%) as compared with negative control (*A. alternata*) after 30, 60, and 90 DAS of seedlings. The application of mycorrhiza alone corresponded to a 31.33% and 26.66%; 42.10% and 57.86%; and 20.76% and 94.18% increase in FW and DW (in the presence of phytopathogens) after 30, 60, and 90 DAS of seedlings, respectively. The plant length and number of leaves increased by 105.03% and 98.55%; 94.55% and 93.22%; 130.34% and 81.22% in treatments that received P-WBF along with mycorrhiza as compared to negative control after 30, 60, and 90 DAS of seedlings, respectively (Table 1). In the presence of phytopathogen (*A. alternata*), the plant length and number of leaves of *S. rebaudiana* treated with P-WBF (only) increased by 67.51% and 86.15%; 71.42% and 66.95%; and 65.22% and 65.51%, respectively, after 30, 60, and 90 DAS of seedlings. The number of leaves and plant length were also enhanced by the treatment of mycorrhiza (only) when it was compared with negative control. Overall, the results suggest that a combined treatment of P-WBF and mycorrhiza was the best method for the enhancement of the number of leaves, plant length, FW, and DW of *S. rebaudiana* as compared to other treatments performed in the study.

### 2.3. Impact of Bioformulations on Biochemical Aspects of S. rebaudiana

**Figure 2:**
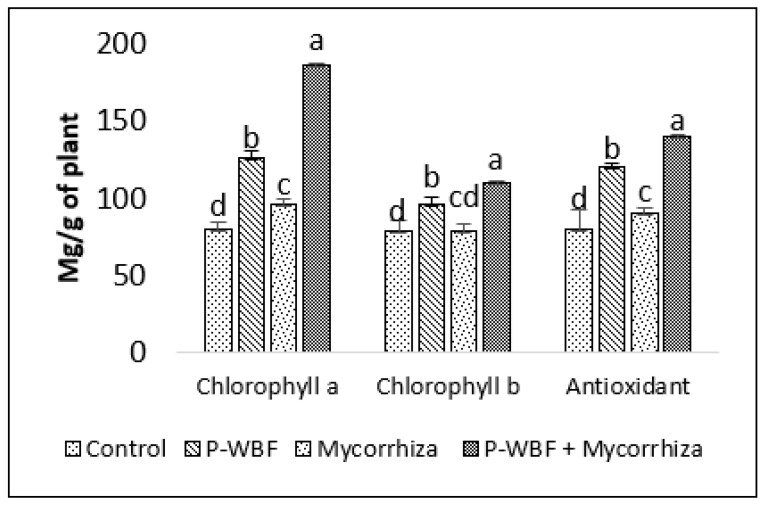
Chlorophyll content and antioxidant activity in plants after application of P-WBF and mycorrhiza (90 DAS of seedlings). Data are mean of three replicates ± standard error of means. Different letters in each parameter indicate significant difference DMRT (*p* ≤ 0.05).

*S. rebaudiana* grown in the field showed higher chlorophyll a (57.71%) and chlorophyll b (22.31%) content when treated with P-WBF even in the presence of phytopathogens/control. A significant increase in chlorophyll content (a and b) was observed after a combined treatment of P-WBF and mycorrhiza (132.85% and 39.80%) as compared to control. Although the plants treated with mycorrhiza alone also increased chlorophyll content (a and b), their result was not as good as compared to other treatments (Figure 2). 

**Figure 3:**
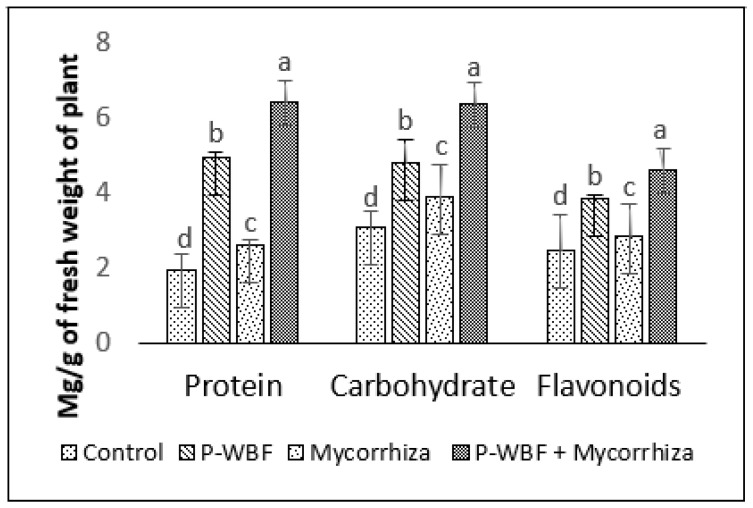
Flavonoid, carbohydrate, and protein content in plants after application of P-WBF and mycorrhiza (90 DAS of seedlings). Data are mean of three replicates ± standard error of means. Different letters in each parameter indicate significant difference by DMRT (*p* ≤ 0.05).

There was a 278.75%, 86.99%, and 75.11% enhancement in protein, flavonoid, and antioxidant activity in the plants treated with P-WBF and mycorrhiza, in comparison with control. P-WBF and mycorrhiza (separately) also improved the protein (153.06% and 33.67%), flavonoid (56.91% and 16.26%), and antioxidant activity (50.39% and 12.63%) (in the plants) in the presence of phytopathogens, but it was significantly less than the P-WBF and mycorrhiza treatment in combination (Figure 2 and Figure 3).

Mycorrhiza-, P-WBF-, and P-WBF plus mycorrhiza-treated plants resulted in a higher carbohydrate content by 25.64%, 50.39%, and 103.84%, respectively, as compared with control (Figure 3). The protein content improved by 227.55% as compared to control by the combined treatment of P-WBF and mycorrhiza (Figure 3). Overall, the results suggest that P-WBF and mycorrhiza combined was the best treatment for the enhancement of antioxidant activity and chlorophyll, carbohydrate, and flavonoid contents.

### 2.4. Stevioside Content

**Figure 4:**
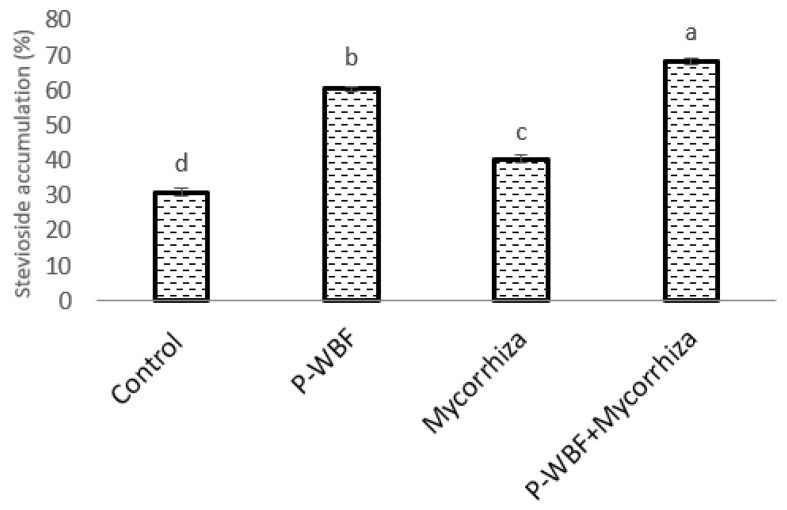
Effect of P-WBF and mycorrhiza treatment on stevioside content in *S. rebaudiana* (90 DAS of seedlings). Data are mean of three replicates ± standard error of means. Different letters in each column indicate significant difference by DMRT (*p* ≤ 0.05).

**Figure 5:**
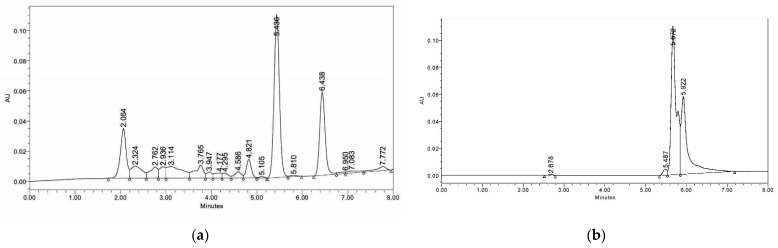
HPLC chromatogram showing stevioside in control plants of *S. rebaudiana* (90 DAS of seedlings) (**a**). HPLC chromatogram showing stevioside by *S. rebaudiana* after P-WBF and mycorrhiza treatment (90 DAS of seedlings) (**b**).

In the presence of *A. alternata*, the maximum stevioside content (120.62%) in *S. rebaudiana* was obtained under combined treatment with P-WBF and mycorrhiza (Figure 4). P-WBF-treated plants had significantly enhanced stevioside content (95.39%) when compared with control. However, this result was not significant in comparison to the combined treatment (P-WBF and mycorrhiza). Mycorrhiza-treated plants also had enhanced stevioside content (29.97%) as compared to the negative control (*A. alternata*). Overall, the results suggest that the combined treatment of P-WBF and mycorrhiza was a more effective method for the augmentation of stevioside content in *S. rebaudiana*. This result was compared and confirmed with the chromatogram of stevioside (under different treatments) by high-performance liquid chromatography (HPLC) (Figure 5a,b).

### 2.5. Impact of P-WBF and Mycorrhiza on Nutrient Uptake in S. rebaudiana

The combined treatment of P-WBF and mycorrhiza resulted in a 94.22% enhancement of phosphorus (P) content in the plant when compared with control (Table 2). P-WBF and mycorrhiza (individually) positively affected the P content as compared to uninoculated plants and resulted in a 71.52% and 24.39% increment in the uptake of P, respectively, as compared to control (*A. alternata*). Zinc (Zn) uptake in *S. rebaudiana* was also significantly augmented when applied with P-WBF plus mycorrhiza (111.11%) in comparison with the control plants (Table 2). The amount of Zn content was increased by 93.70% and 48.14% with P-WBF and mycorrhiza treatments (separately) in comparison to uninoculated plants, respectively. P-WBF along with mycorrhiza significantly enhanced potassium (K) (132.39%) content in comparison with the control plants (Table 2). Mycorrhiza- and P-WBF-treated plants (separately) showed a 34.57% and 87.22% increment in the uptake of K when compared with the control plants. Overall, the combined treatment (P-WBF plus mycorrhiza) was found to be better for the uptake of nutrients (P, K, and Zn) by *S. rebaudiana*.

### 2.6. Quantitative Analysis of UGT85C2, UGT74G1, and UGT76G1 Genes in Steviol Glycoside Biosynthesis

**Figure 6:**
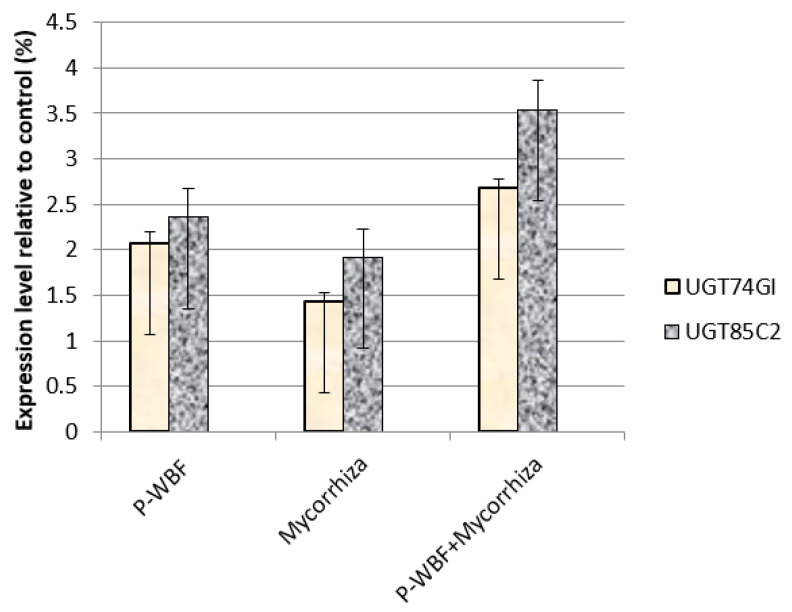
Transcript accumulation profiling of candidate genes in SG biosynthesis by P-WBF and mycorrhiza treatment (Data are mean of standard deviation with three replicates).

In the current experiment, the relative gene expression related to SG biosynthesis was modified with the application of different treatments (Figure 6). P-WBF-treated plants showed upregulation of the UGT85C2 and UGT74G1 genes, resulting in enhanced stevioside and steviolmonoside production (results show 2.2- and 2.8-fold enhancement); on the other hand, gene UGT76G1 was downregulated, decreasing the production of rebaudioside-A. In addition, mycorrhiza also upregulated the expression of UGT85C2 and UGT74G1 while downregulating the expression of UGT76G1. The combined application of mycorrhiza plus P-WBF showed a strong transcriptional profile in SG production. Except for the UGT76G1 gene, the expression of the UGT85C2 and UGT74G1 genes was upregulated (3.22- and 2.7-fold), which caused significant enhancement in the steviolmonoside and stevioside content (Figure 6). Overall, the combined treatment (mycorrhiza plus P-WBF) was found to be more effective in the modification of SG biosynthesis.

### 2.7. Impact of Bioformulation on Soil Health

**Figure 7:**
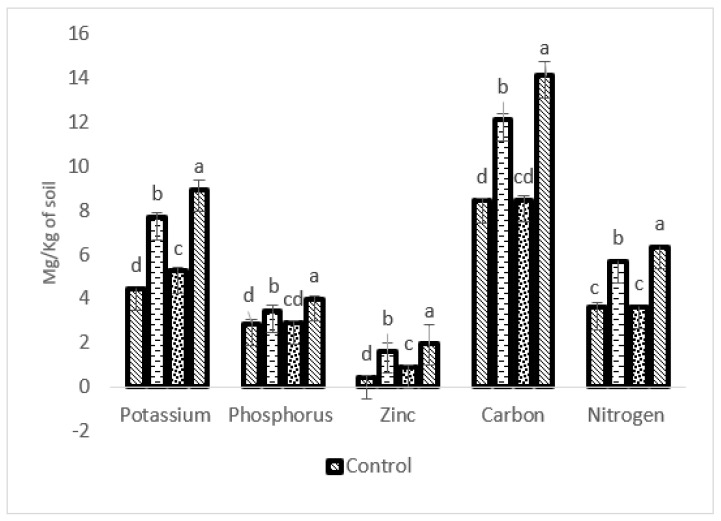
Nutrient content in soil after application of P-WBF and mycorrhiza. Data are mean of three replicates ± standard error of means. Different letters in each parameter indicate significant difference by DMRT (*p* ≤ 0.05).

After the harvesting of *S. rebaudiana*, the nutrients in the soil were checked, and it was observed that K, P, Zn, C, and N of the combined treatment (P-WBF and mycorrhiza)-treated soils were 100.89, 39.16, 366.66, 67.10, and 74.72% higher, respectively, than without any treatment (Figure 7). Soil treated with P-WBF increased K, P, Zn, C, and N at the levels of 72.19, 20.97, 80.95, 43.66, and 56.31%, respectively, in comparison with control (Figure 7). Mycorrhiza-treated soil was 17.48, 2.09 and 40.25% higher in K, P, and Zn content, respectively, in the presence of phytopathogen (*A. alternata*). However, mycorrhiza on its own did not significantly affect the C and N content when the result was compared with control (Figure 7). The experimental field soil was alkaline (pH: 8.3) in the control set. In this regard, the pH of all the treated soils was recorded between 6.7 and 7.8 (P-WBF and mycorrhiza: 6.9; P-WBF: 7.2; and mycorrhiza: 7.8). Overall, the P-WBF and mycorrhiza inoculation was found to be a good method for the improvement of soil fertility. The effects of the inoculation (P-WBF and mycorrhiza) resulted in a significant impact on soil quality and plant productivity.

## 3. Discussion

PGPR have been extensively used for the enhancement of plant growth and control of phytopathogens [24]. The application of PGPR can be a sustainable approach to manage leaf spot disease (caused by *A. alternata*) in *S. rebaudiana*. In the current study, isolate STJP produced 16.80 µL of salicylic acid in in vitro conditions. Salicylic acid is a natural phenolic compound and plant growth hormone in many plants, and its role is considered in the growth promotion of plants and improvement in soil quality by the prevention of fungal phytopathogens via inducing systemic resistance mechanisms [25]. Salicylic acid produced by *B. amyloliquefaciens* CNU114001 accelerated mineral solubilization in soil and inhibited the growth of *Rhizoctonia solani* and *Alternaria* sp. [26]. El-Garhy et al. [27] demonstrated that exogenous applications of salicylic acid on tomato crops enhanced induced systematic resistance (ISR) against *A. alternata* and improved its quality and productivity.

*B. safensis* STJP showed biocontrol against *A. alternata*, and its CFS was also effective against the phytopathogen. The bacterium strain STJP produced chitinase (75.58 U/mL), a cell wall (fungal)-degrading enzyme belonging to the glycosyl hydrolase family, and mainly secreted by rhizobacteria [28]. This enzyme can enhance the nutrient mobilization and the plant’s defense system against fungal phytopathogens due to the degradation of chitin present in the fungal cell wall, and its role is further reported in the augmentation of growth and productivity of plants [29]. Many rhizobacteria, including *Bacillus* spp., use chitin as an energy source by degrading it with the help of chitinase [30]. The isolate STJP also produced β-1, 3-glucanase (220.36 U/mL) along with cellulase. These enzymes have the ability to break down the glycosidic bond in the cell wall of fungal phytopathogens and also help in the upgrading of soil fertility, cell division, seed maturation, rapid growth, flower formation, and mobilization of nutrients in plant tissue [28,31]. The *Bacillus halodurans* strain C-125 inhibited fungal growth and soil nutrients by the production of β-1, 3-glucanase and cellulase enzyme [32]. Overall, the isolate STJP was found to be a very good PGPR because of its ability to produce different plant growth-promoting (PGP) metabolites, such as indole 3-acetic acid (IAA), siderophore, solubilized potassium, phosphate, and zinc [1,20], and it also produced biocontrol metabolites, such as salicylic acid, chitinase, and β, 1,3-glucanase. Such isolates can be very important for use as a bioinoculant. In this regard, strain STJP (*B. safensis* NAIMCC-B-02323) was used to develop paneer whey-based bioformulation (P-WBF). Based on the shelf life, ease of availability, and presence of nutrients, it was found to be an effective liquid carrier (paneer whey) [1], and hence it was further applied for field trial.

Better plant biomass (fresh and dry weight), number of leaves, plant length, and nutrient uptake was observed in *S. rebaudiana* by the treatment of P-WBF and mycorrhiza in the presence of the fungal pathogen, which might be associated with the solubilization of minerals and the production of hormones, siderophores and lytic enzymes by *B. safensis* STJP. A cocktail of PGP properties (plant growth and biocontrol traits) of *Bacillus* spp. will help translocate minerals by the nutrient transpiration xylem pathway [33] and increase plant productivity by the growth inhibition of fungal pathogens, such as *A. alternata* [26,34]. The bioformulation developed utilizing *Bacillus coagulans* applied on a sugar beet crop, showed high biomass and yield of the plant due to the solubilization of P, K, and Zn in the presence of phytopathogens [35]. Furthermore, the mutualistic interaction between mycorrhizal fungi and plants is one of the most important biological interactions involved in the augmentation of plant growth under nutrient-deficient and naturally infested soils. The mycorrhizal nutrient solubilization of Zn, K, and P directly aids in the increase in fresh biomass and crop productivity [23].

In the presence of phytopathogens, the combined treatment (P-WBF and mycorrhiza) increased chlorophyll, carbohydrate, flavonoid, and antioxidant activity in *S. rebaudiana* as compared to other treatments performed in the study. *Bacillus*-based bioformulations with several PGP properties have been found to improve plant growth and nutrient, chlorophyll, and metabolite content in a variety of medicinal, vegetable, and fruit crops [36,37]. The *Bacillus* sp. strain CL12 enhanced the growth parameters, including flavonoid and antioxidant activity and metabolite (curcumin) content, in *Curcuma longa* due to the solubilization of nutrients and the production of hormones, siderophore, and lytic enzymes [38]. *Bacillus* spp. are also reported to control leaf spot disease (*A. alternata*) of *Aloe vera* [39] and also improved biochemical aspects and metabolite contents of the plants [38,39]. Similarly, *B. safensis* STJP-based bioformulation displayed PGP properties, and its application on the stevia plants increased growth parameters, biochemical aspects, and metabolite contents in the presence of the fungal pathogen. 

Stevioside is a key sweetener found in the stevia plant and is a sugar alternative. In the present study, the combined treatment of P-WBF and mycorrhiza gave the highest levels of stevioside content (120.53% as compared to control) under phytopathogen-infested conditions. This might be due to the production of various PGP traits including biocontrol activity against *A. alternata*. The combined application of *Bacillus* sp. with mycorrhiza showed better growth, disease management, and yield of the coriander plant by nutrient translocation as compared to a single application (*Bacillus* or mycorrhiza) or without any treatment in the field [40]. The combined treatment (P-WBF plus mycorrhiza) was found to be better for the nutrient (P, K, and Zn) uptake by *S. rebaudiana*. However, in the present study, the number of leaves, plant length, FW, DW, photosynthetic pigments (chlorophylls), and biochemical aspects were significantly reduced in the control sets. Furthermore, the nutrient uptake in the stevia plant was low in comparison with the treated sets. Due to all these factors, there was a reduced metabolite (stevioside) content in the control sets. Overall, the developed P-WBF utilizing *B. safensis* STJP was capable of controlling leaf spot disease in stevia and promoted growth, yield, and stevioside accumulation. Until now, there have been no reports on the bioformulation for *S. rebaudiana* growth enhancement and secondary metabolite augmentation at the field scale. According to our knowledge, this is the first study on the application of P-WBF to increase growth and stevioside content in *S. rebaudiana* in *A. alternata*-infested fields.

Medicinal and aromatic plants (MAPs) are well known to produce a number of important secondary metabolites. In the current study on the application of PWF and mycorrhiza, SG biosynthesis was enhanced in *S. rebaudiana* even when grown in an *A. alternata*-infested field. We observed that the UGT74G1 and UGT85C2 genes showed upregulation, while the UGT76G1 gene was found to be downregulated in the stevia plant. Modi et al. [9] observed that among the three genes, the UGT76G1 gene was upregulated in the biosynthesis of SGs, enhancing the rebaudioside-A production after gibberellin treatment. In our study, we found that it was the UGT74G1 gene of the plant rather than the UGT76G1 gene that resulted in modification of the SGs pattern. However, the expression of the UGT76G1 gene might be suppressed in the presence of a fungal pathogen (*A. alternata*), and it showed downregulation and reduced rebaudioside-A accumulation. Moreover, the expression of such genes can be upregulated by the application of chemicals or PGP additives [10,41]. Kumar et al. [42] reported that the UGT74G1 gene was upregulated in the SG biosynthesis pathway when it was treated with gibberellic acid in *S. rebaudiana*. The mycorrhiza-treated plant augmented SG production by the upregulation of UGT74G1 gene expression; the result suggests that the gene improves stevioside production correlating with increased sweetness [31,42]. The co-inoculation of mycorrhiza and PGPR thus resulted in a strong transcriptional profile and improved SG production.

Soil fertility mainly depends on nutrient availability, pH, healthy microbial population, and uptake of soluble nutrients by the roots to promote plant growth and productivity [40]. Soil nutrients, such as P, K, Fe, and Zn, are generally unavailable to the plant because of their insolubility in the soil [43]. In the current study, the combined application of P-WBF and mycorrhiza improved soil pH (6.9) and solubilized nutrients (K, P, Zn, C, and N) resulting in the improvement of nutrient availability in the soil. The pH of the soil is important for the survival of helpful microbes and plant growth. Soil fertility, microbial population, and plant growth can all be affected by pH levels that are too low or too high. Improved soil pH resulted in better availability of nutrients thus significantly impacting plant growth and nutrient (metabolite) content [44,45].

Overall, *B. safensis* STJP showing PGP traits, such as phosphate, Zn, and K solubilization and the ability to chelate Fe (via siderophore) along with biocontrol traits (lytic enzymes and salicylic acid), can be a big boon for the overall management of *S. rebaudiana* cultivation. The combined application of the PGPR and mycorrhiza not only improved plant growth parameters and stevioside content but also controlled the leaf spot disease. This may help in enhancing the quality of produce and will be helpful in improving the production of a very important plant metabolite.

## 4. Materials and Methods

### 4.1. Microorganisms, Phytopathogen, and Seedling Collection

Bacterium strain STJP was isolated from the rhizospheric locale of *S. rebaudiana* plant and identified as *Bacillus safensis* NAIMCC-B-02323 as already reported by the authors [1,20]. Afterward, paneer whey-based bioformulation (P-WBF) using *B. safensis* was developed [1] and taken for field trials. *Glomus fasciculatum* (ABTEC), a vesicular-arbuscular mycorrhiza (VAM) was purchased online from www.amazon.com (accessed on 10 January 2018) (Manufactured by Agro Bio-Tech Research Centre Ltd., Kerela, India). *A. alternata* (accession number-KX494864, NCBI), a phytopathogen of *S. rebaudiana,* was procured from the Laboratory for Rhizospheric Microbiology and Sustainable Agriculture, School for Earth and Environmental Sciences, Babasaheb Bhimrao Ambedkar University, Lucknow, Uttar Pradesh, India. Seedlings (variety: CIM-Mithi) of *S. rebaudiana* were obtained from the Central Institute of Medicinal and Aromatic Plants (CIMAP), Lucknow, Uttar Pradesh, India.

### 4.2. Biocontrol Characteristics of Isolate

For the qualitative and quantitative estimation of hydrogen cyanide, a nutrient agar plate containing glycine was used as per Bakker and Schipper [46]. The quantitative determination of salicylic acid was checked in a succinate medium by the cell-free culture extraction method [47].

Isolate STJP was grown in chitin–peptone medium and centrifuged, and collected supernatant was taken as sample for the estimation of chitinase activity [48]. For estimation of β-1, 3-glucanase, STJP was inoculated in peptone medium amended with laminarin (0.2%, Sigma-Aldrich, St. Louis, Missouri, USA) as per Lim et al. [48]. After incubation, medium was centrifuged, and the supernatant was taken for quantification of β-1, 3-glucanase [28,48]. Cellulase activity was checked on an optimized medium containing carboxymethyl cellulose (CMC) as per Ghose et al. [49].

### 4.3. Antagonistic Activity by Cell Suspension and Cell-Free Supernatant from Isolate

The antagonistic activity of isolate STJP against *A. alternata* was evaluated via CS and CFS as per Soria and Audisio [50]. To obtain the CS and CFS, isolate (1% *v*/*v*) was inoculated in the nutrient broth (HiMedia, Mumbai, India) and incubated in a shaker (150 rpm, 28 °C, and 48 h). After incubation, the growth medium was centrifuged (9000 rpm, 15 min, 4 °C). CFS was collected after centrifugation, and CS was obtained through a sterilized membrane filter (0.22 µm pore-size cellulose acetate membrane) as per Soria and Audisio [50]. For the examination of the antifungal assay, CS and CFS were inoculated into separate potato dextrose broth (PDB) containing *A. alternata* and incubated at 150 rpm, 28 °C, and 120 h. After incubation, the broth was centrifuged, and numbers of cells/spores of fungus were counted as per Soria and Audisio [50].

### 4.4. Field Study

The developed paneer whey-based bioformulation (P-WBF) using *B. safensis* STJP [1] was used for validation in natural (unsterilized soil) field conditions. The field experiment was performed in Horticulture Research Farmhouse (HRF), BBAU, Lucknow (latitude 26.8467° N and longitude 80.9462° E) with an elevation of 123 m above sea level from February to April 2018 (90 days) and repeated from February to April 2019 for 90 days as per Tewari and Arora [51]. During the experiments, temperatures were recorded between 12 °C and 36 °C; humidity was recorded between 46% and 82%, and occasional rainfall was recorded in February. The pH, electrical conductivity, exchangeable sodium percentage, organic content, sand, silt, and clay of the experimental field were determined to be 8.2, 3.2 mmhos cm^−1^, 14 %, 0.8%, 7%, 1.1%, and 84%, respectively. The soil of the field is naturally infested with *A. alternata* (10^3^ CFU/g of soil). The field experiment was designed using a completely randomized block design (CRD) with five replicates of each treatment in a standard plot size of 200 m^2^ (15 m × 10 m), where each block was 2 m × 2 m, with the following treatments: (1) control (*A. alternata*/experimental field), (2) *B. safensis* STJP (bioformulation), (3) mycorrhiza (*Glomus fasciculatum*), and (4) mycorrhiza + *B. safensis* STJP. An approximate 0.20 m distance was left in between each block, and 28-day seedlings of *S. rebaudiana* (variety: CIM-Mithi) of approximately the same size (selected based on visual observation) were sown in a 35 cm row width. Before sowing the seedlings, the CFU of strain STJP was counted (~10^8^ CFU mL^−1^) in the bioformulation using plate count and standard serial dilution methods. The seedling of stevia plant was treated by bacterial strain STJP by root-dip method [20], while mycorrhiza treatment in stevia seedling was performed according to Mandal et al. [52]. During the entire experiment, normal water was used for irrigation (four-fold) in the field. No chemicals (fertilizers or pesticides) were applied in this trial. FW, DW, number of leaves, and root and shoot length were checked 30, 60, and 90 days after planting.

### 4.5. Effect of Bioformulation on Biochemical Aspects of S. rebaudiana

The examination of carbohydrate (soluble sugar) was performed by Anthrone method [53]. The soluble sugar (carbohydrate) was calculated (g^−1^ FW of the leaf by using a standard curve of glucose) as per Albalasmeh et al. [53]. The protein content of stevia leaves was determined in mg/g FW of the leaves according to the standard method of Lowry [54] using bovine serum albumin (BSA) (Sisco-pH 6-7, fraction V) as standard. Chlorophyll content (chlorophyll a and chlorophyll b) of the plant was checked and expressed in mg/g FW of leaf according to Arnon [55]. Antioxidant activity was determined by 2, 2-diphenyl-2-picrylhydrazyl (DPPH) assay using methanol standard as per Braca et al. [56]. For the estimation of flavonoid content, aluminum chloride colorimetric method was used [57] by using quercetin (HPLC grade) (Hi-Media, Mumbai, India) solution in methanol as standard, and the content was expressed in mg per gram DW of the plant as per Quettier-Deleu et al. [57].

### 4.6. Estimation of Stevioside Content

After 90 days of treatment, *S. rebaudiana* shoots were harvested from each treatment and dried at 50 °C in a hot air oven. Furthermore, using the hot water method, stevioside was extracted with water at 78 ± 2 °C for 56 min [58]. The resulted sample was filtered using Whatman filter paper Number 42, and then water was vaporized (45 °C; 55 rpm min^−1^; 75 mbar) through a rotatory evaporator (model: R-100 Thermo Fisher Scientific, USA) as per Rahi et al. [59]. The obtained sample containing stevioside was mixed with acetonitrile and water (both HPLC grade, Merck, Mumbai, India) in a 20:80 ratio. The blended sample was filtered via a millipore membrane (0.45 µm) in high-performance liquid chromatography (HPLC) vial, and the obtained sample was tested for stevioside content using HPLC (Waters 2489 UV/Visible Detector, Milford, MA, USA) entailing mixed-mode wax-1 column (5μm). Afterward, 20 µL dissolved sample was introduced into the sample loop, and a further mobile phase was set in an 80:20 *v*/*v* ratio of acetonitrile and water (HPLC Grade, Merck, Mumbai, India) at the pressure of 1100 psi, flow rate of 1.0 mL/min, run time of 10 min, and temperature of 40 °C as per Rahi et al. [59]. The result was viewed at 210 nm after injecting the sample in an analytical column. The obtained result was interpreted through standard stevioside (98% purity, Sigma-Aldrich, St. Louis, Missouri, USA).

### 4.7. Effect of P-WBF on Nutrient Uptake in Stevia Plants

*S. rebaudiana* plants were harvested (90 DAS of seedlings) and dried in a hot air oven (temp: 50 ± 2 °C; time: 4 days) for nutritional estimation. The dried plant sample (100 mg) was grinded and then digested with H_2_SO_4_ and HClO_4_ (6 mL) in a ratio of 9:1 using the hot plate technique (10 min) to determine phosphorus (P) content. The colorless, digested sample was diluted in double-distilled water (100 mL). The obtained sample was then tested for P using the vanadomolybdophosphoric yellow color technique [60]. The Zn uptake in *S. rebaudiana* was measured following digesting process as per Horwitz et al. [61] using atomic absorption spectroscopy (AAS; model: AA240FS Fast Sequential AAS, Agilent Technologies, San Francisco, USA). The uptake of K content in the plant was estimated with a triacid digestion procedure as per Jackson [62].

### 4.8. Quantitative Analysis of UGT85C2, UGT74G1, and UGT76G1 Genes in Steviol Glycoside Biosynthesis by RT-qPCR

#### 4.8.1. Plant Sample, Primer Designing, and Its Validation

For the investigation of overexpression of candidate genes, same stage, uniform axial-positioned leaves of control and treated plants were taken (after 90 days of growth). Sequences of candidate genes were taken from the model organism, and the primers were designed using Gene Runner Software (version 6) as per Kumar et al. [42]. The major three genes involved in the biosynthesis of SGs were UGT85C2, UGT74G1, and UGT76G1. Primers of the genes were provided to make a stock solution (100 pmol with 0.3× TE buffer), and then this solution was diluted 10 pmol for PCR master mix preparation. For the validation of gene primers, 1.0 µL and 0.2 µL samples (in 25 µL and 20 µL systems) were taken each for DNA confirmation and real-time experiments, respectively [42]. cDNA sample was taken for control and run in RT-PCR using all the selected primers. The steps of PCR (cycling conditions) before the melting curve of designed genes and all validation procedures were followed as per Kumar et al. [42].

#### 4.8.2. RNA Extraction and cDNA Synthesis

For isolation of RNA, TRIzol reagent was used as per Ghawana et al. [63]. Briefly, 50 mg of leaf or tissue and 1 mL of TRIzol reagent (Sigma-Aldrich, St. Louis, Missouri, USA) was mixed for homogenization. Then, 0.2 mL of chloroform was added to lyse the tissues or cells and centrifuged at 13,000 rpm for 10 min. The aqueous phase (upper part of the sample) was mixed with isopropanol (0.6 mL), and further centrifugation was performed to obtain a sample in precipitate form. Afterward, the sample was rinsed with 75% chilled ethanol. The obtained RNA was dissolved in treated water of diethylpyrocarbonate (DEPC) as per Modi et al. [9].

According to the manufacturer’s instructions, the cDNA was primed from cDNA synthesis kit for RT-qPCR (BioAdvanced Systems, Mexico. Briefly, reaction mixtures (20 µL) containing total RNA, dNTP mix, reverse transcriptase, RNase inhibitor, and oligo dT primers were incubated for 60 min at 37 °C followed by 10 min at 65 °C to denature enzyme present in the sample, and the obtained samples were diluted 5 times and used as a working solution [9].

#### 4.8.3. Quantitative Analysis of UGT85C2, UGT74G1, and UGT76G1 Genes in Steviol Glycoside Biosynthesis Using RT-qPCR

The gene expression was tested by real-time quantitative polymerase chain reaction (RT-qPCR) using Act and 18S rRNA genes as endogenous control [42,64]. cDNA synthesis was prepared as described above, and PCR was carried out using 1 µL of cDNA template in a 25 µL reaction. For each gene, conditions of PCR cycling were optimized to achieve amplification beneath the exponential phase of PCR as per Kumar et al. [42]. The samples (control and treatments) were analyzed using SyberGreen mix as fluorophore to quantify by RT-qPCR (Model: BIO-RAD C1000) with three replicates, and the result was interpreted using 2^−ΔΔCT^ Method [9]. Signal intensities were examined, and the image was captured using a gel documentation machine (Model: iBright^TM^ CL1000, Thermo Fisher Scientific, Waltham, Massachusetts, USA). AD-1000 software was used for the calculation of integrated density value (IDV) to examine deviations in the expression of candidate genes [9].

### 4.9. Effect of Bioformulation on Soil Health

After the tilling of plants, soil pH and electrical conductivity were measured by the standard method of Jackson [62]. The total organic carbon in the soil was observed using a wet oxidation method described by Grewal et al. [65]. The available P content in soil (treated and nontreated) was determined by using Kjeldahl digestion method [66]. Total nitrogen (N) from the soil sample was analyzed with an aqueous solution (HCl/HNO_3_) in a ratio of 3:1 as per the alkaline permanganate method [67]. After the physiochemical properties, the biological properties of soil (in each pot) were examined separately. 

### 4.10. Experimental Statistical Analysis

The field experiment used a completely randomized block design (CRD) with five replicates, and the data were reported as the mean minus standard deviation (SD). Analysis of variance (ANOVA) was used to examine the result validity, and Duncan’s multiple range test (p ≤ 0.05) was used to check the significant differences between distinct sets [68]. Three replicates of gene expression related to SG biosynthesis were performed, and the statistical significance of the candidate gene was determined using the ANOVA test (*p* = 0.05) as per Compton [69].

## 5. Conclusions

The use of *B. safensis* as a bioformulation to promote plant growth and manage fungal pathogen is an environmentally friendly method. P-WBF-based *B. safensis* bioformulation has had a significant impact on *S. rebaudiana* growth parameters, biochemical aspects and metabolite attributes, as well as on SG biosynthesis, and has proven to be a viable alternative to chemicals. *B. safensis* has a variety of PGP and biocontrol properties and was used as a novel paneer whey-based bioformulation to boost stevia plant growth and SG accumulation by delivering an array of nutrients. The current study’s findings suggest that *B. safensis* with PGP abilities could be a viable tool for improving farmer livelihoods through improved *S. rebaudiana* cultivation in an ecofriendly manner. Exploiting a PGP strain such as *B. safensis* as a cheap and effective bioformulation could be a better option for soil reclamation, sustainable agriculture, and production of green metabolites for the agro, food, beverage and pharmaceutical industries.

## Figures and Tables

**Table 1:** Plant growth parameters of *S. rebaudiana* after application of P-WBF and mycorrhiza.

Treatments	Growth Parameters of *S. rebaudiana* after Application of P-WBF and Mycorrhiza (30 Days)						
	Plant Length (cm)		Number of Leaves	Fresh Weight (gm)		Dry Weight (gm)	
	Shoot Length	Root Length	-	Shoot	Root	Shoot	Root
Control	13.30 ± 2.28 d	3.20 ± 0.56 d	45.66 ± 2.10 d	20.10 ± 1.24 d	5.24 ± 0.86 d	9.30 ± 0.86 d	2.10 ± 0.04 d
P-WBF	22.50 ± 2.21 b	5.00 ± 1.02 a	85.00 ± 2.14 b	35.24 ± 2.40 b	8.62 ± 0.96 a	12.45 ± 1.24 b	5.00 ± 0.05 b
Mycorrhiza	18.30 ± 2.45 c	5.10 ± 0.28 c	80.42 ± 2.36 c	26.42 ± 1.42 c	6.86 ± 0.80 c	10.24 ± 1.12 c	4.20 ± 0.70 c
P-WBF + Mycorrhiza	28.83 ± 2.04 a	5.14 ± 1.60 ab	90.66 ± 4.12 a	40.12 ± 1.24 a	8.44 ± 0.94 ab	14.20 ± 1.60 a	6.20 ± 1.02 a
	**Growth Parameters of *S. rebaudiana* after Application of P-WBF and Mycorrhiza (60 days)**						
	**Plant Length (cm)**		**Number of Leaves**	**Fresh Weight (gm)**		**Dry Weight (gm)**	
	**Shoot Length**	**Root Length**	**-**	**Shoot**	**Root**	**Shoot**	**Root**
Control	18.08 ± 1.78 d	4.32 ± 0.86 d	85.33 ± 2.84 d	35.72 ± 2.60 d	7.46 ± 0.86 d	15.10 ± 2.20 d	3.98 ± 0.86 d
P-WBF	31.60 ± 4.48 b	6.80 ± 2.46 b	152.46 ± 2.80 b	58.46 ± 2.45 b	10.24 ± 0.48 b	26.10 ± 1.24 b	8.62 ± 0.80 ab
Mycorrhiza	28.63 ± 4.56 c	5.96 ± 0.80 c	142.0 ± 4.24 bc	51.84 ± 2.45 c	9.52 ± 0.48 c	22.50 ± 1.10 c	7.62 ± 0.54 c
P-WBF + Mycorrhiza	36.34 ± 5.45 a	7.24 ± 1.89 a	164.88 ± 5.24 a	62.34 ± 2.47 a	12.54 ± 1.14 a	30.12 ± 3.24 a	8.96 ± 1.21 a
	**Growth Parameters of *S. rebaudiana* after Application of P-WBF and Mycorrhiza (90 days)**						
	**Plant Length (cm)**		**Number of Leaves**	**Fresh Weight (gm)**		**Dry Weight (gm)**	
	**Shoot Length**	**Root Length**	**-**	**Shoot**	**Root**	**Shoot**	**Root**
Control	23.43 ± 4.25 d	5.90 ± 1.14 d	135.44 ± 6.10 d	62.45 ± 2.84 d	9.64 ± 1.12 d	25.36 ± 2.12 d	5.96 ± 0.98 d
P-WBF	40.26 ± 4.15 b	8.20 ± 1.50 b	224.18 ± 6.88 b	118.42 ± 4.42 b	20.56 ± 1.48 b	60.12 ± 3.46 b	12.24 ± 2.24 b
Mycorrhiza	30.12 ± 2.46 c	7.00 ± 0.84 c	180.60 ± 6.50 c	74.52 ± 1.86 cd	12.54 ± 0.84 c	50.24 ± 2.42 c	10.58 ± 1.70 c
P-WBF + Mycorrhiza	58.46 ± 4.46 a	9.1 ± 2.42 a	245.45 ± 5.20 a	142.46 ± 4.86 a	24.89 ± 1.88 a	74.56 ± 4.56 a	14.46 ± 1.12 a

Data are mean of five replicates ± standard error of means. Different letters in each parameter indicate significant difference by Duncan’s multivariate test (DMRT) (*p* ≤ 0.05).

**Table 2:** Nutrient content in *S. rebaudiana* after application of P-WBF and mycorrhiza (90 days).

Treatments	Potassium Content (mg/plant)	Phosphorus Content (mg/plant)	Zinc Content (mg/plant)
Control	3.21 ± 0.07 d	129.01 ± 6.84 d	1.08 ± 0.08 d
P-WBF	6.01 ± 0.41 b	221.28 ± 5.76 b	2.12 ± 0.10 b
Mycorrhiza	4.32 ± 0.63 c	160.48 ± 5.68 c	1.60 ± 0.10 c
P-WBF + Mycorrhiza	7.46 ± 0.30 a	250.57 ± 12.79 a	2.28 ± 0.04 a

Data are mean of five replicates ± standard error of means. Different letters in each parameter indicate significant difference by DMRT (*p* ≤ 0.05).

## Data Availability

Data are available from the authors on request.

## Supplementary Materials

The following supporting information can be downloaded at: https://www.mdpi.com/article/10.3390/plants11141857/s1, Figure S1: Improvement in growth of *S. rebaudiana* by combined application of P-WBF and mycorrhiza 90 DAS of seedlings (in the field)

## References

[1] Prakash J., Arora N.K. (2020). Development of *Bacillus safensis*-based liquid bioformulation to augment growth, stevioside content, and nutrient uptake in *Stevia rebaudiana*. World J. Microbiol. Biotechnol..

[2] Raspe D.T., da Silva C., da Costa S.C. (2022). Compounds from *Stevia rebaudiana* Bertoni leaves: An overview of non-conventional extraction methods and challenges. Food Biosci..

[3] Ali A., Shahu R., Balyan P., Kumari S., Ghodmare R., Jobby R., Jha P. (2022). Antioxidation and antiglycation properties of a natural sweetener: *Stevia rebaudiana*. Sugar Tech..

[4] Latridis N., Kougioumtzi A., Vlataki K., Papadaki S., Magklara A. (2022). Anti-Cancer Properties of *Stevia rebaudiana*; More than a Sweetener. Molecules.

[5] Gaweł-Bęben K., Bujak T., Nizioł-Łukaszewska Z., Antosiewicz B., Jakubczyk A., Karaś M., Rybczyńska K. (2015). *Stevia* rebaudiana Bert. leaf extracts as a multifunctional source of natural antioxidants. Molecules.

[6] Azzam C.R., Al-Taweel S.K., Abdel-Aziz R.M., Rabea K.M., Abou-Sreea A.I., Rady M.M., Ali E.F. (2021). Salinity effects on gene expression, morphological, and physio-biochemical responses of stevia rebaudiana bertoni in vitro. Plants.

[7] Ahmad N., Rab A., Sajid M., Ahmad N., Fazal H., Ali M., Egertsdotter U. (2021). Sucrose-dependent production of biomass and low-caloric steviol glycosides in adventitious root cultures of *Stevia rebaudiana* (Bert.). Ind. Crops Prod..

[8] Lee S.Y., Shaari K. (2022). LC–MS metabolomics analysis of *Stevia rebaudiana* Bertoni leaves cultivated in Malaysia in relation to different developmental stages. Phytochemical Anal..

[9] Modi A., Litoriya N., Prajapati V., Rafalia R., Narayanan S. (2014). Transcriptional profiling of genes involved in steviol glycoside biosynthesis in *Stevia rebaudiana* bertoni during plant hardening. Develop. Dynam..

[10] Vazquez-Hernandez C., Feregrino-Perez A.A., Perez-Ramirez I., Ocampo-Velazquez R.V., Rico-García E., Torres-Pacheco I., Guevara-Gonzalez R.G. (2019). Controlled elicitation increases steviol glycosides (SGs) content and gene expression-associated to biosynthesis of SGs in *Stevia rebaudiana* B. cv. Morita II. Ind. Crops Prod..

[11] Ahmad A., Ali H., Khan H., Begam A., Khan S., Ali S.S., Abbasi B.H. (2020). Effect of gibberellic acid on production of biomass, polyphenolics and steviol glycosides in adventitious root cultures of Stevia rebaudiana (Bert.). Plants.

[12] Sarmiento-López L.G., López-Meyer M., Sepúlveda-Jiménez G., Cárdenas L., Rodríguez-Monroy M. (2020). Photosynthetic performance and stevioside concentration are improved by the arbuscular mycorrhizal symbiosis in Stevia rebaudiana under different phosphate concentrations. Peer J..

[13] Bandyopadhyay P., Yadav B.G., Kumar S.G., Kumar R., Kogel K.H., Kumar S. (2022). *Piriformospora indica* and *Azotobacter chroococcum* Consortium Facilitates Higher Acquisition of N, P with Improved Carbon Allocation and Enhanced Plant Growth in *Oryza sativa*. J. Fungi.

[14] Mandal P., Tiru Z. (2022). Soil Application of Plant Growth Promoting Fungi for Sustainable Agriculture in the New Decade. Plant Stress: Challenges and Management in the New Decade.

[15] Prakash J., Arora N.K. (2021). Novel metabolites from *Bacillus safensis* and their antifungal property against *Alternaria alternata*. Antonie Van Leeuwenhoek.

[16] Meena M., Swapnil P., Upadhyay R.S. (2017). Isolation, characterization and toxicological potential of *Alternaria*-mycotoxins (TeA, AOH and AME) in different *Alternaria* species from various regions of India. Sci. Rep..

[17] Bashan Y., Prabhu S.R., de-Bashan L.E., Kloepper J.W. (2020). Disclosure of exact protocols of fermentation, identity of microorganisms within consortia, formation of advanced consortia with microbe-based products. Biol. Fertil. Soils.

[18] Mishra J., Prakash J., Arora N.K. (2016). Role of beneficial soil microbes in sustainable agriculture and environmental management. Climate Change Environ. Sustain..

[19] Prakash J., Arora N.K. (2019). Phosphate-solubilizing *Bacillus* sp. enhances growth, phosphorus uptake and oil yield of *Mentha arvensis* L. 3 Biotech..

[20] Wang S., Sun L., Zhang W., Chi F., Hao X., Bian J., Li Y. (2020). *Bacillus velezensis* BM21, a potential and efficient biocontrol agent in control of corn stalk rot caused by *Fusarium graminearum*. Egypt. J. Biolog. Pest Contr..

[21] Gao X., Guo H., Zhang Q., Guo H., Zhang L., Zhang C., Gou Z., Liu Y., Wei J., Chen A. (2020). Arbuscular mycorrhizal fungi (AMF) enhanced the growth, yield, fiber quality and phosphorus regulation in upland cotton (*Gossypium hirsutum* L.). Sci. Rep..

[22] Bennett A.E., Groten K. (2022). The Costs and Benefits of Plant–Arbuscular Mycorrhizal Fungal Interactions. Annu. Rev. Plant Biol..

[23] Pickles B.J., Truong C., Watts-Williams S.J., Bueno C.G. (2020). Mycorrhizas for a sustainable world. New Phytol..

[24] Singh P., Singh R.K., Zhou Y., Wang J., Jiang Y., Shen N., Jiang M. (2022). Unlocking the strength of plant growth promoting *Pseudomonas* in improving crop productivity in normal and challenging environments: A review. J. Plant Inter..

[25] Kang S.M., Radhakrishnan R., Lee I.J. (2015). *Bacillus amyloliquefaciens* subsp. plantarum GR53, a potent biocontrol agent resists *Rhizoctonia* disease on Chinese cabbage through hormonal and antioxidants regulation. World J. Microbiol. Biotechnol..

[26] El-Garhy H.A., Abdel-Rahman F.A., Shams A.S., Osman G.H., Moustafa M.M. (2020). Comparative analyses of four chemicals used to control black mold disease in tomato and its effects on defense signaling pathways, productivity and quality traits. Plants.

[27] Kumar M., Brar A., Yadav M., Chawade A., Vivekanand V., Pareek N. (2018). Chitinases-potential candidates for enhanced plant resistance towards fungal pathogens. Agriculture.

[28] Wang C., Thielemann L., Dippold M.A., Guggenberger G., Kuzyakov Y., Banfield C.C., Dorodnikov M. (2022). Can the reductive dissolution of ferric iron in paddy soils compensate phosphorus limitation of rice plants and microorganisms?. Soil Biol. Biochem..

[29] Kumar M., Dangayach P., Pareek N. (2020). Enhanced glucosamine production through synergistic action of *Aspergillus terreus* chitozymes. J. Cleaner Prod..

[30] Behera B.C., Sethi B.K., Mishra R.R., Dutta S.K., Thatoi H.N. (2017). Microbial cellulase–Diversity & biotechnology with reference to mangrove environment: A review. J. Gen. Eng. Biotechnol..

[31] Chen Y., Xu H., Zhou M., Wang Y., Wang S., Zhang J. (2015). Salecan enhances the activities of β-1, 3-glucanase and decreases the biomass of soil-borne fungi. PLoS ONE.

[32] Mia M.A. (2022). Enhanced Root Morphogenesis in Non-legumes as Induced by *Rhizobacteria Bacillus* spp. Bacilli in Agrobiotechnology.

[33] Abdelmoteleb A., Troncoso-Rojas R., Gonzalez-Soto T., González-Mendoza D. (2017). Antifungical activity of autochthonous *Bacillus subtilis* isolated from *Prosopis juliflora* against phytopathogenic fungi. Mycobiology.

[34] Jorjani M., Heydari A., Zamanizadeh H.R., Rezaee S., Naraghi L. (2011). Development of *Pseudomonas fluorescens* and *Bacillus coagulans* based bioformulations using organic and inorganic carriers and evaluation of their influence on growth parameters of sugar beet. J. Biopest..

[35] Farhaoui A., Adadi A., Tahiri A., El Alami N., Khayi S., Mentag R., Lahlali R. (2022). Biocontrol potential of plant growth-promoting rhizobacteria (PGPR) against *Sclerotiorum rolfsii* diseases on sugar beet (*Beta vulgaris* L.). Physiol. Mol. Plant Pathol..

[36] Sardar H., Nisar A., Anjum M.A., Naz S., Ejaz S., Ali S., Ahmad R. (2021). Foliar spray of moringa leaf extract improves growth and concentration of pigment, minerals and stevioside in stevia (*Stevia rebaudiana* Bertoni). Ind. Crops Prod..

[37] Kumar A., Singh M., Singh P.P., Singh S.K., Singh P.K., Pandey K.D. (2016). Isolation of plant growth promoting rhizobacteria and their impact on growth and curcumin content in *Curcuma longa* L. Biocat. Agri. Biotech..

[38] Ghosh S.K., Banerjee S., Pal S., Chakraborty N. (2018). Encountering epidemic effects of leaf spot disease (*Alternaria brassicae*) on *Aloe vera* by fungal biocontrol agents in agrifields: An ecofriendly approach. PLoS ONE.

[39] Mangwende E., Kritzinger Q., Aveling T.A.S. (2019). Control of *Alternaria* leaf spot of coriander in organic farming. Eur. J. Plant Pathol..

[40] Gorain B., Paul S., Parihar M. (2022). Role of soil microbes in micronutrient solubilization. New and Future Developments in Microbial Biotechnology and Bioengineering.

[41] Zheng J., Zhuang Y., Mao H.Z., Jang I.C. (2019). Overexpression of SrDXS1 and SrKAH enhances steviol glycosides content in transgenic *Stevia* plants. BMC Plant Biol..

[42] Kumar H., Kaul K., Bajpai-Gupta S., Kaul V.K., Kumar S. (2012). A comprehensive analysis of fifteen genes of steviol glycosides biosynthesis pathway in *Stevia rebaudiana* (Bertoni). Gene.

[43] Nieves-Cordones M., Martínez V., Benito B., Rubio F. (2016). Comparison between Arabidopsis and rice for main pathways of K+ and Na+ uptake by roots. Front. Plant Sci..

[44] Saleem S., Khan S.T. (2022). Development of Microbes-Based Biofertilizer for Zinc Dissolution in Soil. Microbial Biofertilizers and Micronutrient Availability.

[45] Prakash J., Arora N. (2021). Novel Metabolites Identified From *Bacillus Safensis* and Their Antifungal Property Against *Alternaria Alternata*. Antonie Van Leeuwenhoek.

[46] Bakker A.W., Schippers B. (1987). Microbial cyanide production in the rhizosphere in relation to potato yield reduction and *Pseudomonas* spp-mediated plant growth-stimulation. Soil Biol. Biochem..

[47] Meyer J.A., Abdallah M.A. (1978). The fluorescent pigment of *Pseudomonas fluorescens*: Biosynthesis, purification and physicochemical properties. Microbiology.

[48] Lim H., Kim Y., Kim S. (1991). *Pseudomonas stutzeri* YLP-1 genetic transformation and antifungal mechanism against *Fusarium solani*, an agent of plant root rot. Appl. Environ. Microbiol..

[49] Ghose T.K., Montenecourt B.S., Eveleigh D.E. (1987). Commission of biotechnology, measurement of cellulase activities. Pure Appl. Chem..

[50] Soria M.C., Audisio M.C. (2014). Inhibition of *Bacillus cereus* strains by antimicrobial metabolites from *Lactobacillus johnsonii* CRL1647 and *Enterococcus faecium* SM21. Probio. Anti. Prot..

[51] Tewari S., Arora N.K. (2014). Multifunctional exopolysaccharides from *Pseudomonas aeruginosa* PF23 involved in plant growth stimulation, biocontrol and stress amelioration in sunflower under saline conditions. Cur. Microbiol..

[52] Mandal S., Upadhyay S., Singh V.P., Kapoor R. (2015). Enhanced production of steviol glycosides in mycorrhizal plants: A concerted effect of arbuscular mycorrhizal symbiosis on transcription of biosynthetic genes. Plant Physiol. Biochem..

[53] Albalasmeh A.A., Berhe A.A., Ghezzehei T.A. (2013). A new method for rapid determination of carbohydrate and total carbon concentrations using UV spectrophotometry. Carbohydr. Poly..

[54] Lowry O.H., Rosebrough N.J., Farr A.L., Randall R.J. (1951). Protein measurement with the Folin phenol reagent. J. Biolog. Chem..

[55] Arnon D.I. (1949). Copper enzymes in isolated chloroplasts: Polyphenoloxidase in Beta vulgaris. Plant Physiol..

[56] Braca A., De Tommasi N., Di B.L., Pizza C., Politi M., Morelli I. (2001). Antioxidant principles from bauhinia t arapotensis. J. Natur. Prod..

[57] Quettier-Deleu C., Gressier B., Vasseur J., Dine T., Brunet C., Luyckx M., Cazin M., Cazin J.C., Bailleul F., Trotin F. (2000). Phenolic compounds and antioxidant activities of buckwheat (*Fagopyrum esculentum* Moench) hulls and flour. J. Ethnopharmacol..

[58] Rai C., Majumdar G.C., De S. (2012). Optimization of process parameters for water extraction of stevioside using response surface methodology. Sep. Sci. Technol..

[59] Rahi P., Pathania V., Gulati A., Singh B., Bhanwra R.K., Tewari R. (2010). Stimulatory effect of phosphate-solubilizing bacteria on plant growth, stevioside and rebaudioside-A contents of *Stevia rebaudiana* Bertoni. Appl. Soil Ecol..

[60] Koenig R., Johnson C. (1942). Colorimetric determination of phosphorus in biological materials. Ind. Eng. Chem. Anal..

[61] Horwitz W., Chichilo P., Reynolds H. (1970). Official methods of analysis of the association of official *analytical chemists*. J. Am. Oil Chem. Society.

[62] Jackson M.L. (1967). Soil Chemical Analysis.

[63] Ghawana S., Paul A., Kumar H., Kumar A., Singh H., Bhardwaj P.K., Rani A., Singh R.S., Raizada J., Singh K. (2011). An RNA isolation system for plant tissues rich in secondary metabolites. BMC Res. Notes.

[64] Radonić A., Thulke S., Mackay I.M., Landt O., Siegert W., Nitsche A. (2004). Guideline to reference gene selection for quantitative real-time PCR. Biochem. Biophy. Res. Commun..

[65] Grewal K.S., Buchan G.D., Sherlock R.R. (1991). A comparison of three methods of organic carbon determination in some New Zealand soils. J. Soil Sci..

[66] Taylor M.D. (2000). Determination of total phosphorus in soil using simple Kjeldahl digestion. Commun. Soil Sci. Plant Anal..

[67] Subbiah B.V., Asija G.L. (1956). A rapid method for the estimation of nitrogen in soil. Cur. Sci..

[68] Gomez K.A., Gomez A.A. (1984). Duncan’s multiple Range test. Statistical Proced. Agri. Res..

[69] Compton M.E. (1994). Statistical method suitable for the analysis of plant tissue culture data. Plant Cell Tissue Organ Cult..

