# Supplementary material for: A Novel Bacillus safensis-Based Formulation along with Mycorrhiza Inoculation for Controlling Alternaria alternata and Simultaneously Improving Growth, Nutrient Uptake, and Steviol Glycosides in Stevia rebaudiana under Field Conditions
Source: Plants (Basel). 2022 Jul 15;11(14):1857. doi: 10.3390/plants11141857 (PMC9317049; doi:10.3390/plants11141857)
Supplement: Supplementary file 1 [file plants-11-01857-s001.zip › plants-1769057-supplementary.pdf]

Figure S1. Improvement in growth of *S. rebaudiana* by combined application of P-WBF and mycorrhiza 90 DAS of seedlings (in the field)

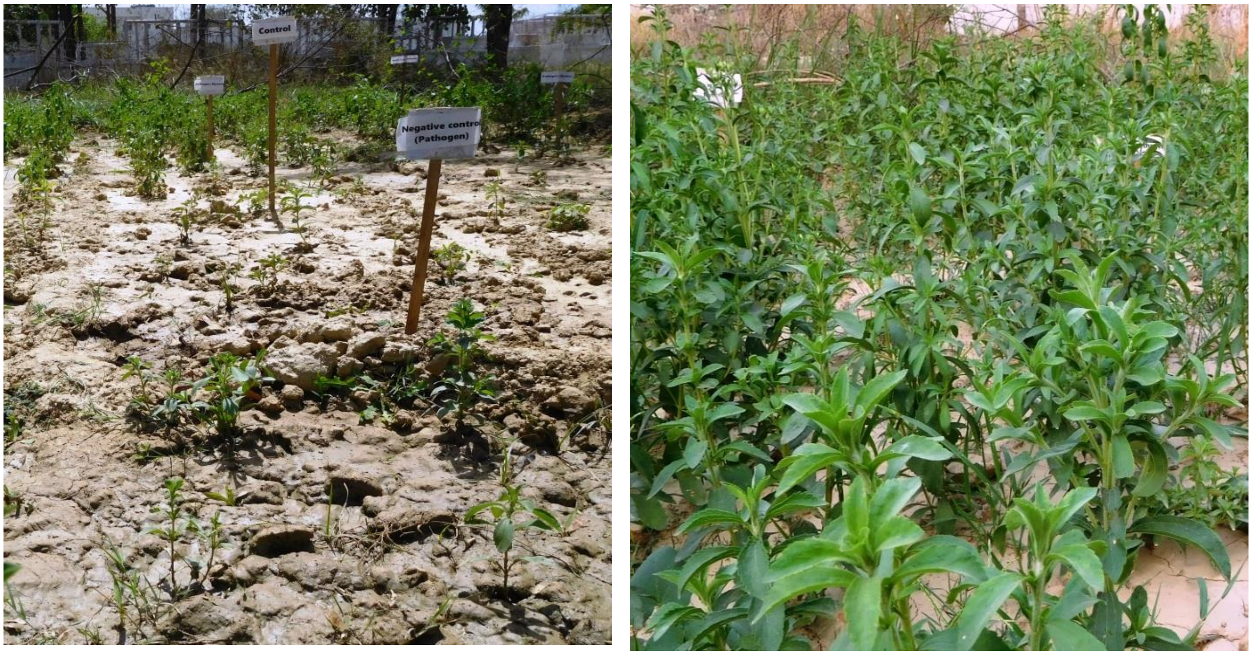

Control in *A. alternata* infested soil 90 DAS of seedling

P-WBF plus mycorrhiza treated set 90 DAS of seedling
